# Real-World Evidence on the Routine Use, Efficacy, and Safety of a Hyaluronic Acid-Based Dermal Filler in the Periorbital Region

**DOI:** 10.1007/s00266-025-04809-9

**Published:** 2025-05-29

**Authors:** Tahera Bhojani-Lynch, Sabrina Shah-Desai, Jean-Christophe Bichet, Bárbara Magalhães, Kevin Poupard

**Affiliations:** 1 The Laser and Light Clinic, Loughborough, UK; 2 Perfect Eyes Ltd., London, UK; 3 Department of Gynecological and Breast Surgery and Oncology, Pitié-Salpêtrière University Hospital, Paris, France; 4 Clinical and Medical Affairs Department, Teoxane S.A., Geneva, Switzerland

**Keywords:** Aesthetic, Facial rejuvenation, Registry, Hyaluronic acid, Dermal filler

## Abstract

**Background:**

The appearance of the periorbital region is essential in facial aesthetic perception and is a frequent concern of patients seeking rejuvenation. TEOSYAL® PureSense Redensity 2 (R2) has a 10-year track record of effectively and safely treating the under-eye area, specifically the tear trough.

**Methods:**

A prospective, observational study aimed to investigate the efficacy and safety of R2 in a real-world setting for aesthetic indications. Participants received at least one treatment injection with R2, and investigators followed their routine injection techniques and indications. The primary endpoint was the Global Aesthetic Improvement Scale (GAIS) score recorded 3 months post-injection. Secondary efficacy outcomes included subject and investigator satisfaction, as well as GAIS scores at later timepoints. Safety was monitored by documenting common treatment responses (CTRs) and adverse events (AEs).

**Results:**

The 136 subjects enrolled in EYELIGHT received 958 initial injections, of which 451 (47.1%) were performed with R2: 89 (35.3%) tear trough, 61 (24.2%) palpebromalar groove, 45 (17.9%) outer canthus, 38 (15.1%) crow’s feet, and 19 (7.5%) brow. A GAIS improvement of more than 70% was achieved for the tear trough and remaining periorbital indications, with most subjects reporting satisfaction with treatment (84.3%). Treatment effect was observed up to 12 months post-injection. All CTRs were mild or moderate and resolved within a month. No severe AEs were reported.

**Conclusion:**

Real-world evidence confirmed the 10-year long success of R2 as an effective and safe treatment of the tear trough. Based on this evidence, its use extends beyond the tear trough, showing effectiveness and safety in the whole periorbital area.

**Level of Evidence III:**

This journal requires that authors assign a level of evidence to each article. For a full description of these Evidence-Based Medicine ratings, please refer to the Table of Contents or the online Instructions to Authors www.springer.com/00266.

## Introduction

The periorbital region holds significant importance in how facial aesthetics are perceived, as it plays a crucial role in communicating emotion through facial expression and stands as a key indicator of the aging process of the face [1–3]. Moreover, it significantly contributes to how an individual’s attractiveness is perceived [4]. Almost 80% of those seeking minimally invasive treatments express a desire to enhance the appearance of their periorbital area [5].

Among the treatment options available, hyaluronic acid (HA) dermal fillers have become increasingly popular as an effective, non-surgical solution for improving the appearance of the periorbital region [6, 7]. The popularity of HA as a filler may be attributed to its immediate results, overall safety and tolerability, non-permanent integration in the soft tissues, and the possibility to be quickly dissolved by hyaluronidase in the case of serious adverse events or undesirable cosmetic outcomes [8]. The rheological properties of HA dermal fillers, such as strength, cohesivity and malleability or stretch, vary considerably depending on the HA concentration, molecular weight [9], degree of modification (cross-linking), and the technology used for modification during manufacturing [10]. The characteristics of the HA filler, as well as its capacity for tissue integration, can significantly impact the outcomes of a procedure in terms of safety and appearance [11]. Consideration should, therefore, be given to anatomical specificities of the target area when selecting an injectable product.

TEOSYAL® PureSense Redensity 2 (TEOXANE, Geneva, Switzerland; R2) has been used for over a decade to treat under-eye circles, with proven efficacy and safety in addressing tear trough concerns [12, 13]. The properties of the gel include low strength, low rigidity, and a low resistance to compression, all of which contribute to its high malleability and spreadability [12, 13]. The incorporation of both crosslinked and non-cross-linked high molecular weight HA, at a low HA concentration, minimizes water absorption and thereby reduces product-associated swelling, which is undesirable in this delicate area of the face [12, 13]. These specific characteristics establish R2 as the product of choice to enhance the under-eye area, particularly the tear trough.

The collection of insights from worldwide practitioners and monitoring of product usage through post-marketing surveillance activities detected the frequent clinical use of R2 beyond the tear trough, specifically in the whole periorbital region (unpublished data). These findings prompted the development of a prospective observational study, EYELIGHT, to gather real-world evidence on the efficacy, safety, and routine use of R2 for aesthetic treatments, including those performed in the periorbital area. The registry was modeled on the GRADUAL study [14].

## Material and Methods

### Study Design and Population

The EYELIGHT study was a multicenter prospective observational study conducted in Europe and designed to demonstrate the performance and safety of R2 in aesthetic treatments. Patients underwent treatment between May 2020 and February 2021 across two centers in the UK and one in France, with data collected until July 2022.

Eligible subjects were those over 18 years old, willing to undergo non-surgical aesthetic procedures and having given their consent. Subjects were excluded from the study if they (I) had been injected with a dermal filler in the same area within the six months preceding the study, (II) presented with a cutaneous disorder, inflammation or infection near to the treatment site, (III) had known hypersensitivity to lidocaine and/or local anesthetic agents or hyaluronic acid, (IV) had autoimmune or cardiac disease, and/or were undergoing treatment for a heart disease, (V) suffered with hepatocellular insufficiency and/or were undergoing treatment for liver disease, (VI) suffered with epilepsy or porphyria, (VII) were undergoing or planning to undergo peeling treatment or laser-/ultrasound-based treatment within 3 weeks following injection, or (VII) were pregnant or breastfeeding.

All enrolled subjects received a minimum of one injection of R2. Treatments for a single indication or for multiple indications were allowed. Any indication could be treated with a single product or a combination of products and could be retreated at any time throughout the duration of the study. The area to be treated, product choice, and injection characteristics—volume, depth, and technique—were all left to the discretion of the practitioner. The EYELIGHT results presented in this publication focus only on R2 used alone to treat periorbital indications (i.e., tear trough, palpebromalar groove, crow’s feet, outer canthus, and brow).

**Fig. 1:**
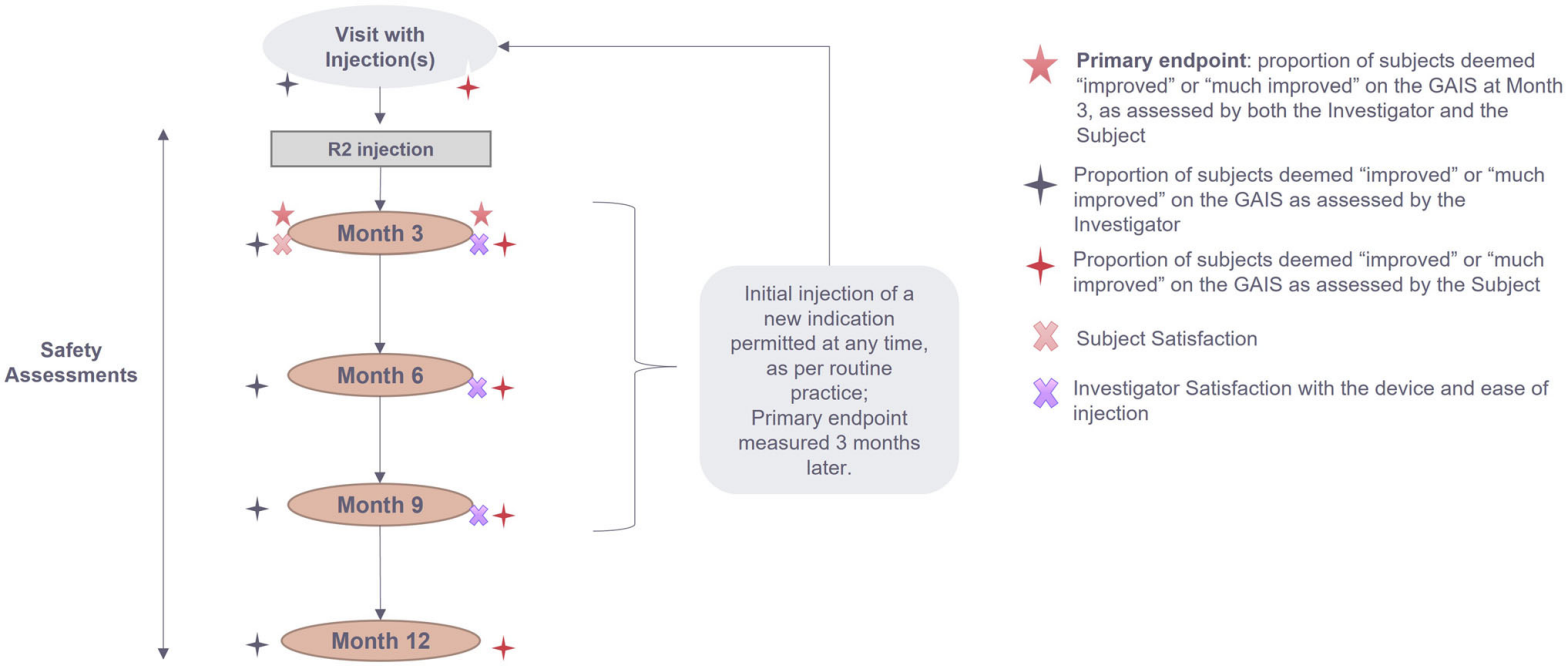
EYELIGHT study design, adapted from the GRADUAL study^14^

The study protocol included an initial visit (D0) and follow-up visits at 3, 6, 9, and 12 months, which were optional as per the observational study design. Additional visits were allowed at any time throughout the follow-up period. Each visit included efficacy and satisfaction assessments for all indications initially treated with R2, safety assessments for all treatments performed during any study visit and could include any type of additional injection: touch-up or retreatment of a previously treated indication, or initial injection in a new area (Figure 1).

Written feedback from the Medicines and Healthcare Regulatory Agency (MHRA, UK) and the French Ethics Committee (CPP) confirmed that the EYELIGHT study did not require ethics approval. The primary investigator (PI) of each participating site ensured that the study was conducted in compliance with the Declaration of Helsinki and national regulations applicable to Good Clinical Practice. Informed consent adhered to specific country regulatory requirements. Image rights were obtained via informed consent.

### Outcomes and Measurements

#### Global Aesthetic Improvement Scale

The Global Aesthetic Improvement Scale (GAIS) was used to evaluate aesthetic improvement of each periorbital indication compared to the appearance before initial injection. As per the study protocol, an initial injection was defined as the first treatment of a specific indication, including touch-ups received by a subject having signed an informed consent form; an initial injection can be received later than Visit 1. Moreover, the term 'total injections' encompasses the treatment of each individual indication, including initial injections and touch-ups, as well as subsequent treatments in other periorbital indications. The appearance is scored on a scale of 1–5, with 1 being ‘much improved’ and 5 being ‘much worse.’ The aesthetic improvement assessment was conducted directly after injection and subsequently every 3 months until month 12.

#### Satisfaction Surveys

Subject satisfaction was surveyed at month 3 after the initial treatment of each indication. The first two questions of the survey asked the subject to rate their level of satisfaction with the treatment and asked how likely they would be to recommend the HA dermal filler products on a 6-point scale. The third and final question asked which word they would best associate with the treatment result: ‘natural looking,’ ‘improved,’ ‘smoothed’ or ‘no opinion.’ Investigator satisfaction was surveyed using two yes/no questions after each injection, until month 9 after the first injection received by the subject. The two questions were: ‘Was the product easy to inject?’ and ‘Are you satisfied with this product?’.

#### Safety

Common treatment responses (CTRs) were collected immediately after injection by the investigator and through an eDiary filled in daily by the subject, for 1 month after each injection. The subject was asked to complete the diary by documenting the listed effects that commonly occur after injection of dermal fillers: redness, pain, tenderness, swelling, firmness, lumps, bruising, itching, discoloration, and other local reactions.

All adverse events (AEs) were assessed by the investigator. The severity of any AE was recorded as mild (i.e., symptoms are barely noticeable or do not make the subject uncomfortable; the AE does not influence performance of daily activities; prescription drugs are not ordinarily needed for symptom(s) relief), moderate (i.e., symptoms are of sufficient severity to make the subject uncomfortable; performance of daily activities is influenced; treatment of symptom(s) with prescription drugs or therapies may be needed), or severe (i.e., symptoms are of sufficient severity to cause the subject severe discomfort; performance of daily activities is compromised; treatment of symptom(s) with prescription drugs or therapies may be needed). Details of seriousness, duration, and relationship to the device were also recorded. The severity of each CTR was also recorded as mild, moderate, or severe. CTRs were not classed as AEs unless their severity and/or duration was in excess of that normally observed.

A 100-mm Visual Analog Scale (VAS) was used to measure the subject’s self-assessment of pain immediately after injection and at the end of each injection visit.

#### Study Endpoints

The primary effectiveness endpoint of the study was the proportion of subjects graded as either ‘improved’ or ‘much improved’ on the GAIS at month 3 (± 4 weeks) post-treatment, as assessed by both the investigator and the subject. The primary endpoint was only considered for the initial injection per indication, regardless of other retreatments or touch-ups. To be considered effective, the lower bound of the bilateral 95% confidence interval (CI) was required to be ≥ 70%. The analysis was conducted across all indications pooled together.

If the GAIS score, as assessed by both the investigator and the subject, was missing at month 3, the primary endpoint was considered missing for the main analysis. To account for missing data, a sensitivity analysis was performed whereby the time window for the GAIS score was extended to 29–153 days post-treatment. If the score was still missing beyond this period, the GAIS score recorded directly following injection was used.

Secondary effectiveness endpoints included improvement on the GAIS scale at months 6, 9, and 12 or until the end of the study, as assessed by the investigator and/or the subject, as well as investigator and subject satisfaction.

Safety endpoints were collected for all injected products and for each injection, including retreatments and touch-ups.

## Results

### Subject Demographics

A total of 136 subjects were enrolled in the study over the 9-month period and 129 (94.9%) completed the study. Of the 136 subjects, 122 (89.7%) were female. The mean age at inclusion was 48.4 ± 12.4 years and ranged from 23 to 77 (Table 1). Most subjects were type II (40.7%) or III (32.6%) on the Fitzpatrick scale.

**Table 1:** Subject demographics in the EYELIGHT study

Variable	*N*	Total		
Gender	136	Female Male		122 (89.7%) 14 (10.3%)
Age (mean ± sd / min.; max.)	136	48.4 ± 12.4 / 23 ; 77		
BMI in kg/m^2^ (mean ± sd)	133	23.3 ± 3.6		
Fitzpatrick skin type	135	Type I Type II Type III Type IV Type V Type VI	10 (7.4%) 55 (40.7%) 44 (32.6%) 21 (15.6%) 5 (3.7%) 0 (0.0%)	

SD, standard deviation; Min, minimum; Max, maximum

Of the all included subjects (AIS) population, 113 (83.1%) completed the month 3 (± 28 days) visit, 100 (73.5%) completed the month 6 (± 28 days) visit, 95 (69.9%) completed the month 9 (± 28 days) visit, and 100 (73.5%) completed the month 12 (± 28 days) visit.

### Treatment

**Fig. 2:**
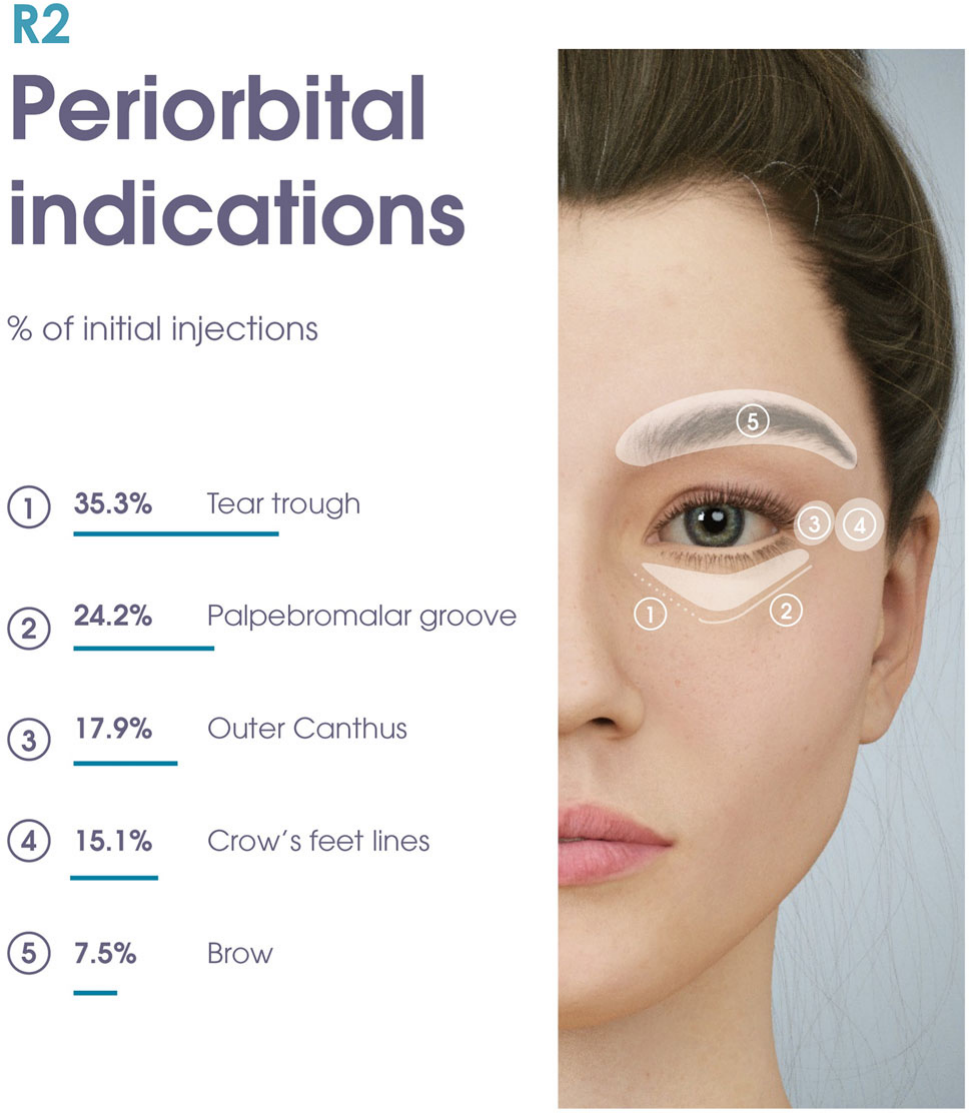
Distribution of main product indications based on the proportion of injections performed in each periorbital area (%), out of all initial treatments performed with R2 alone

Overall, 958 injections were performed at first visit, of which 451 (47.1%) were conducted using R2 alone. Of the initial injections performed with R2 alone in the periorbital area (n = 252, 56%), 89 (35.3%) targeted the tear trough, 61 (24.2%) the palpebromalar groove, 45 (17.9%) the outer canthus, 38 (15.1%) the crow’s feet, and 19 (7.5%) the brow (Table 2, Fig. 2). Most of the injections in the tear trough, palpebromalar groove, and outer canthus were deep, aiming the product to the supraperiosteal plane. Injections in the crow’s feet and brow were more often superficial, targeting either the dermal, subdermal, or subcutaneous planes. The average treatment volume of R2 used to treat one indication was 0.32 ± 0.25 mL, ranging from 0.1 to 1.0 mL (Table 2). Higher volumes of 0.55 ± 0.37 mL (total for both sides) were used in the tear trough. Most treatments in the periorbital area were performed without pre-procedure anesthesia, except 38 (31.7%) injections in the tear trough that used topical anesthetic. The needle provided in the box was used for all but 12 injections. Ten (8.3%) injections in the tear trough and two (2.4%) in the palpebromalar groove were performed using either a cannula or an alternative needle.

**Table 2:** Treatment exposure in the EYELIGHT study

Total number of included subjects	136
Safety analysis (all injections, products and indications)	1376
Efficacy analysis (initial injections performed with R2 alone)	451

Treatment exposure (R2 only)	
Average volume per subject and visit (mean ± sd)	0.52 ± 0.54 mL
Average volume per indication (mean ± sd)	0.32 ± 0.25 mL

Treated indications (R2 only)		
Indication	Initial injections (*n*, % of all periorbital initial injections)	All injections (*n*, % of all periorbital injections)
Tear trough	89 (35.3%)	115 (37.2%)
Palpebromalar groove	61 (24.2%)	78 (25.2%)
Crow’s feet	38 (15.1%)	43 (13.9%)
Outer canthus	45 (17.9%)	53 (17.2%)
Brow	19 (7.5%)	20 (6.5%)

SD, standard deviation

Of the 136 subjects who received an initial injection with R2, only 24 (17.6%) required a touch-up injection while 12 (8.8%) required retreatment.

### Primary Effectiveness Analysis

Of the 451 initial injections with R2 alone, 283 had a GAIS score at month 3. Of these, 214 (75.6%, 95% CI [70.6%; 80.6%]) treatments were graded as ‘improved’ or ‘much improved’ by both the investigator and the subject, across all indications. Given that the lower bound of the 95% CI was greater than 70%, the primary effectiveness endpoint was met.

Considering the sensitivity analysis, 434 initial injections had an available GAIS score either directly post-injection or at 29–153 days post-treatment and could also be included in the primary endpoint analysis. Across all indications, 355 injections (81.8%, 95% CI [78.2%; 85.4%]) were graded as ‘improved’ or ‘much improved’ by both the investigator and the subject, again indicating the achievement of the primary endpoint.

**Fig. 3:**
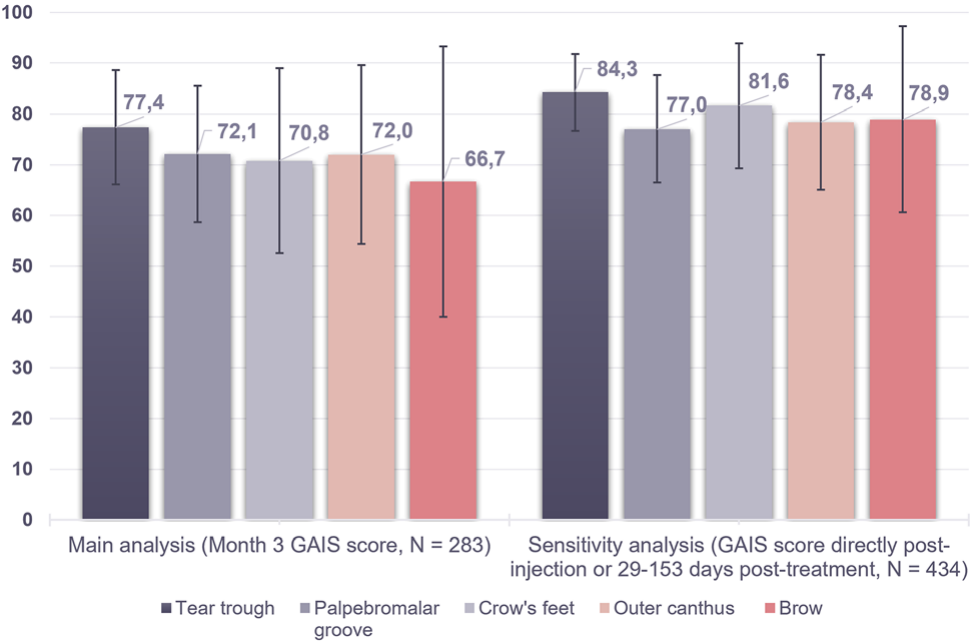
Primary efficacy endpoint (main and sensitivity analyses)

When the main analysis was performed per indication, the tear trough, palpebromalar groove, crow’s feet, and outer canthus exhibited an average improvement in GAIS score exceeding 70% (Table 3, Fig. 3). Once the sensitivity analysis was applied, the brow also reached an average of 70% improvement, meaning that all periorbital indications show substantial improvement on the GAIS score at 3 months post-injection (Table 3, Fig. 3).

**Table 3:** Primary efficacy endpoint by periorbital indication. Proportion of ‘improved’ or ‘much improved’ GAIS scores for periorbital indications as assessed by both investigator and subject

Main analysis (month 3 GAIS score, *n* = 157)			Sensitivity analysis (GAIS score at 29-153 days post-treatment or directly post-injection, *n* = 244)		
Indication	Sample size	‘Improved’ or ‘much improved’ (*n*, %)	Indication	Sample size	‘Improved’ or ‘much improved’ (*n*, %)
Tear trough	53	41 (77.4%)	Tear trough	89	75 (84.3%)
Palpebromalar groove	43	31 (72.1%)	Palpebromalar groove	61	47 (77.0%)
Crow’s feet	24	17 (70.8%)	Crow’s feet	38	31 (81.6%)
Outer canthus	25	18 (72.0%)	Outer canthus	37	29 (78.4%)
Brow	12	8 (66.7%)	Brow	19	15 (78.9%)

### Secondary Effectiveness Analysis

**Fig. 4:**
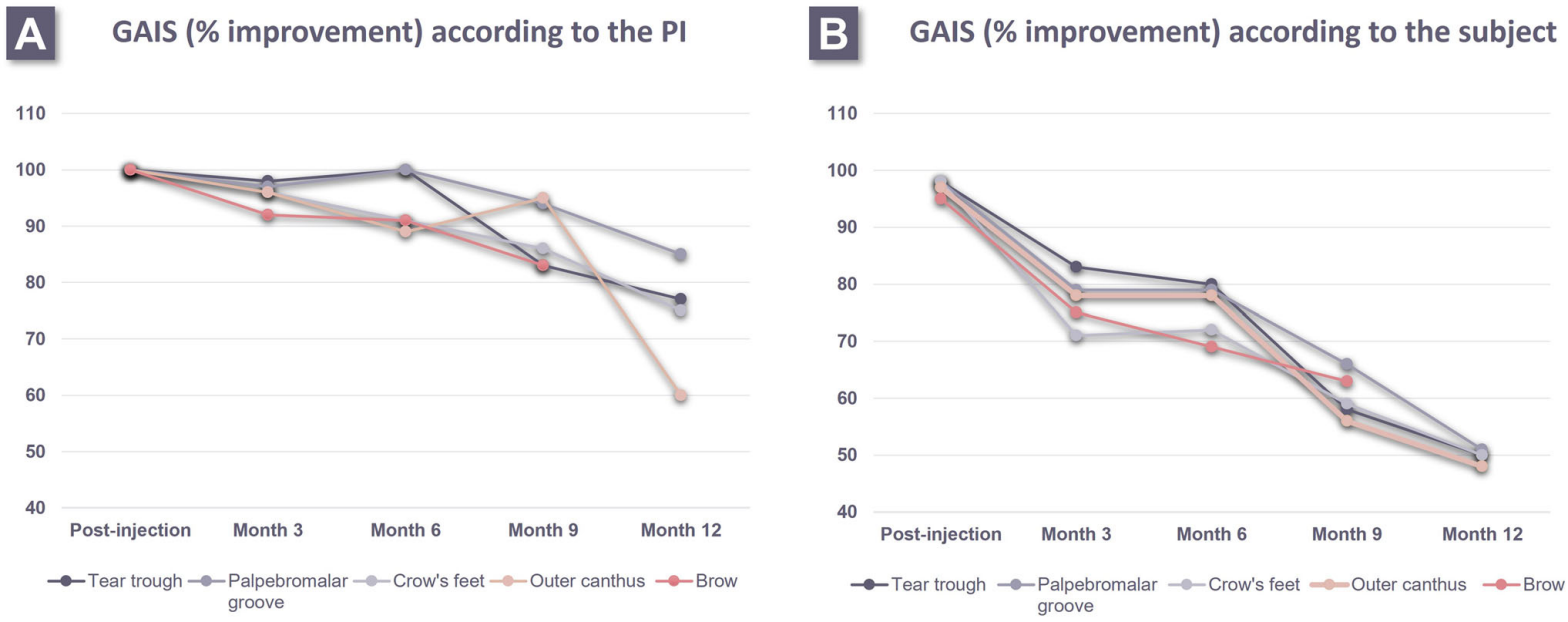
GAIS scores provided by either the PI (**A**) or the subject (**B**) throughout the study period, evaluating the aesthetic improvement in each periorbital indication, as compared to baseline

The proportion of ‘improved’ or ‘much improved’ GAIS scores, as assessed by investigator or subject, was the highest immediately after injection (Fig. 4). Investigator assessments were consistently higher than subject assessments at every timepoint. All subjects were deemed improved post-injection by the investigator for all indications, with at least 82% showing improvement at month 9 and 60% at month 12 (Fig. 4A). The tear trough was the highest rated area by the subject until month 6 (74%) (Fig. 4B). Subject-rated aesthetic improvement was still demonstrated up to month 9 (56–66%) and month 12 (48–51%). Among all indications, the palpebromalar groove had the highest rated improvement by both the investigator (85%) and subject (51%) at 12 months. There were no data collected on the brow at month 12 post-injection.

**Fig. 5:**
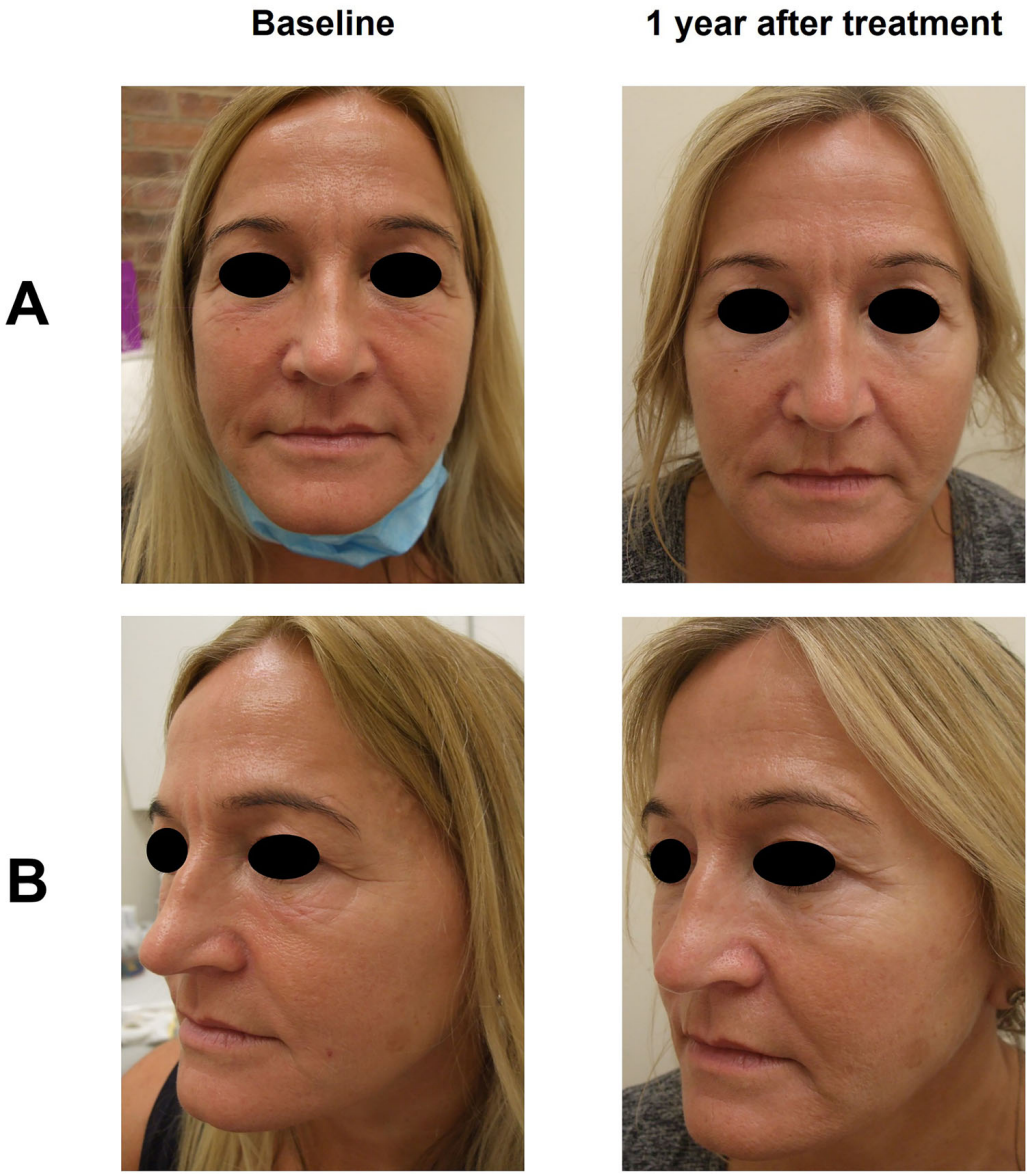
Subject 1. Before and after photographs of a mature female subject who participated in the EYELIGHT study from a frontal (**A**) and left oblique (**B**) view, at baseline and 1 year after treatment. They received R2 to treat the tear trough, palpebromalar groove, outer canthus, and crow’s feet. The subject was also treated for glabellar lines, lip fullness, perioral lines, lip contour, oral commissures, cheek volume, marionette lines, and chin

**Fig. 6:**
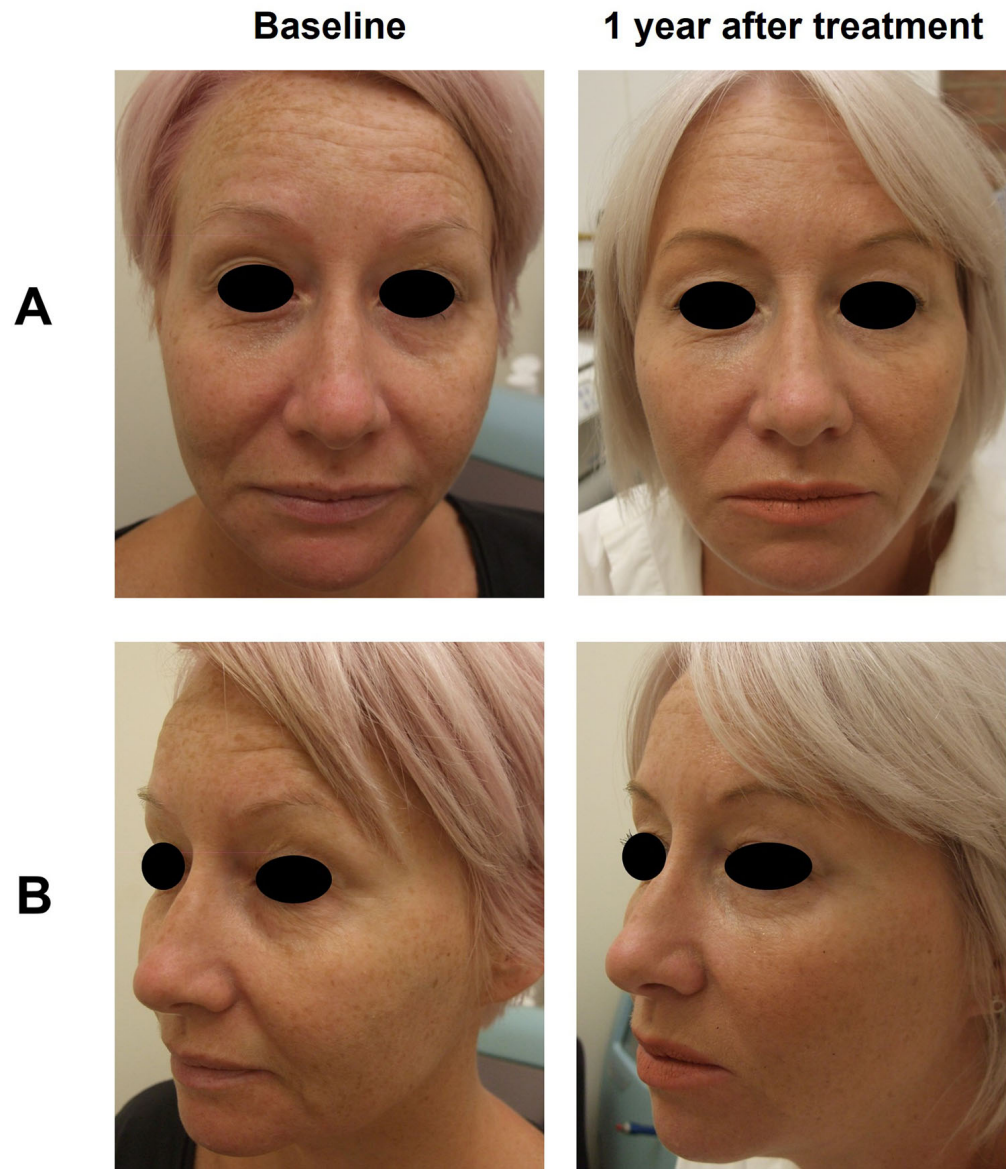
Subject 2. Before and after photographs of a mature female subject who participated in the EYELIGHT study from a frontal (**A**) and left oblique (**B**) view, at baseline and 1 year after treatment. They received R2 to treat the tear trough, palpebromalar groove, outer canthus, and crow’s feet. The subject was also treated for lip fullness, lip contour, marionette lines, forehead lines, glabellar lines, temples, nasojugal groove, and jawline

**Fig. 7:**
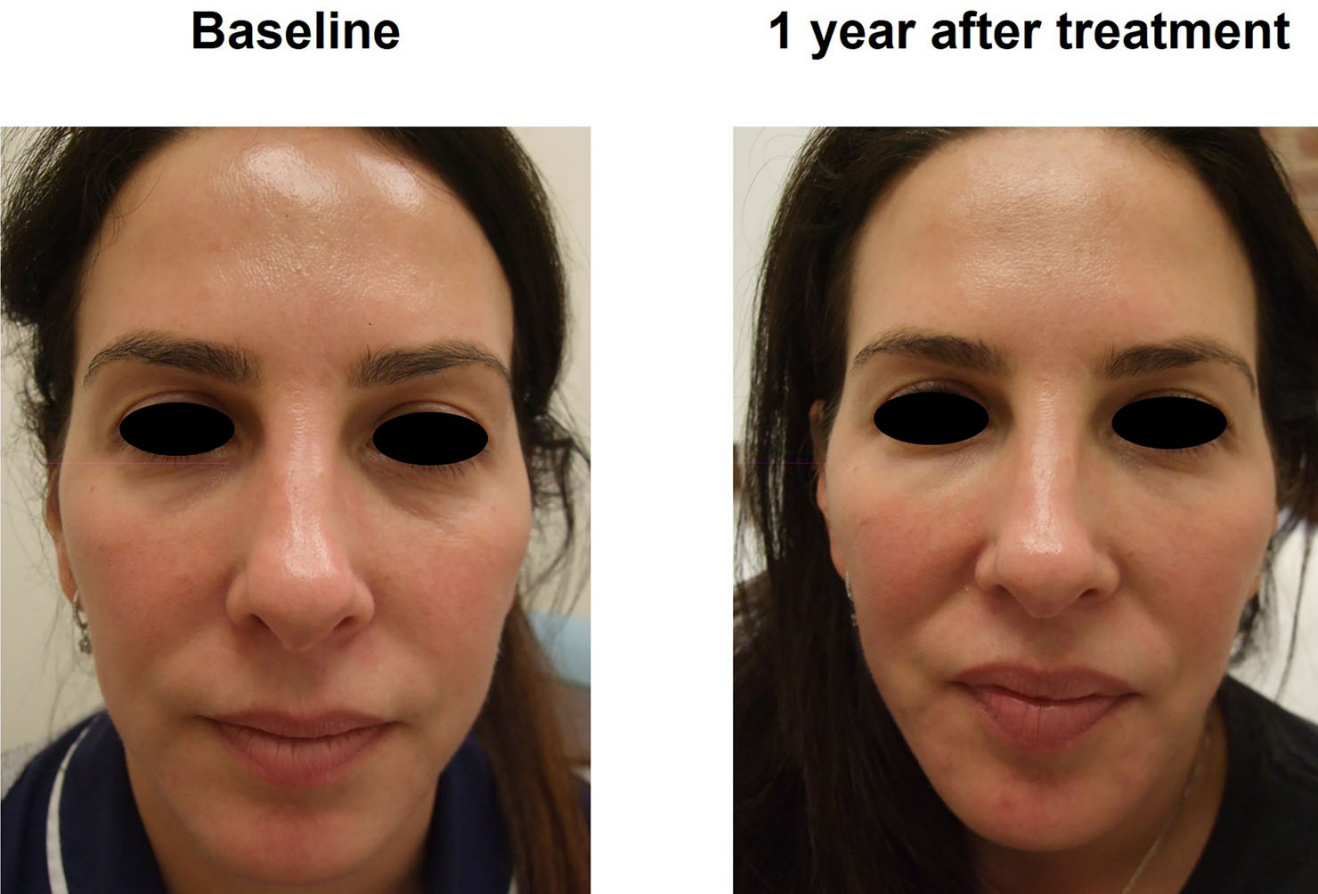
Subject 3. Before and after photographs of a female subject who participated in the EYELIGHT study from a frontal view, at baseline and 1 year after treatment. They received R2 to treat both the tear trough and the outer canthus. The subject was also treated for midface, pyriform fossa, temples, chin, labiomental crease, and jawline

Representative pre-treatment and 12 months after treatment subject images are shown in Figs. 5, 6 and 7.

Subject satisfaction was evaluated at month 3 post-injection for all indications except the brow. A total of 85% subjects were either ‘satisfied’ or ‘very satisfied’ with treatment in the tear trough, 83% in the palpebromalar groove, 92% in the outer canthus, and 69% in the brow. Nearly all subjects were ‘somewhat likely’ or ‘very likely’ to recommend R2 for a similar treatment in the tear trough (93%), palpebromalar groove (83%) and outer canthus (95%), while 69% were ‘somewhat likely’ or ‘very likely’ to recommend R2 treatment in the brow. Most subjects (71% for tear trough; 72% for palpebromalar groove; 74% for outer canthus; 63% for brow) associated their treatment with the word ‘natural looking,’ followed by the words ‘rejuvenated’ (22% for tear trough; 17% for palpebromalar groove; 23% for outer canthus; 19% for brow) and ‘smoothed’ (4% for tear trough; 7% for palpebromalar groove; 3% for outer canthus; 6% for brow).

Almost all investigators were ‘satisfied’ with R2 treatment in the periorbital area (99%), and all investigators (100%) found the device 'easy to inject.'

### Safety

Mean pain felt during injection was between 20 and 30 mm on the 100 mm VAS for all periorbital indications, indicating mild pain. However, in a few subjects, VAS scores reached 80 mm in the tear trough. By the end of the consultation, pain had decreased for all subjects, with mean VAS scores approaching zero (Table 4).

**Table 4:** Injection site pain and common treatment reactions reported by the investigator

		Tear trough	Palpebromalar groove	Crow’s feet	Outer canthus	Brow
VAS during injection	*n*	113	78	43	52	20
	Mean ± sd	20.3 ± 18.7	26.1 ± 15.7	24.8 ± 15.6	26.7 ± 16.3	22.8 ± 15.4
VAS at end of visit	*n*	114	78	43	52	20
	Mean ± sd	0.4 ± 2.2	0.3 ± 1.8	0.1 ± 0.8	0.1 ± 0.7	0.0 ± 0.0
Common treatment reactions	*n*	116	78	48	53	20
	Mild bruising Mild swelling Mild redness Mild lumps and bumps Moderate redness	17 (14.7%) 9 (7.8%) 5 (4.3%) 1 (0.9%) 1 (0.9%)	7 (9.0%) 2 (2.6%) 0 (0.0%) 0 (0.0%) 1 (1.3%)	5 (10.4%) 1 (2.1%) 0 (0.0%) 7 (14.6%) 1 (2.1%)	6 (11.3%) 1 (1.9%) 0 (0.0%) 1 (1.9%) 1 (1.9%)	2 (10.0%) 4 (20.0%) 0 (0.0%) 1 (5.0%) 0 (0.0%)

SD, standard deviation; VAS, Visual Analog Scale

All of the CTRs reported by the investigator immediately after injection were deemed mild or moderate (Table 4). The most frequent reactions in the periorbital region overall were bruising (between 9 and 15%) and swelling (2 and 20%). Of the 116 injections with R2 in the tear trough, 17 (14.7%) resulted in bruising, 9 (7.8%) in swelling, 6 (5.2%) in redness, and 1 (0.9%) in lumps and bumps. Of the 78 injections in the palpebromalar groove, there were 7 (9.0%) cases of bruising, 2 (2.6%) cases of swelling, and 1 (1.3%) case of moderate redness. Out of the 48 injections in the crow’s feet, there were 7 (14.6%) cases of lumps and bumps, 5 (10.4%) cases of bruising, 1 (2.1%) case of moderate redness, and 1 (2.1%) case of swelling. Of the 53 outer canthus treatments, 6 (11.3%) resulted in bruising, 1 (1.9%) resulted in redness, 1 (1.9%) resulted in lumps and bumps, and 1 (1.9%) resulted in swelling. Of the 20 injections in the brow, there were 4 (20.0%) cases of swelling, 2 (10.0%) cases of bruising, and 1 (5.0%) case of lumps and bumps.

Subject eDiaries on CTRs were only filled in by participants at one site, where the only treated indication was the tear trough. Out of 38 injections, 10 (26.3%) resulted in bruising, 4 (10.5%) in lumps and bumps, 4 (10.5%) in swelling, 2 (5.3%) in itching, 1 (2.6%) in pain, 1 (2.6%) in tenderness, 1 (2.6%) in firmness, and 1 (2.6%) in discoloration and were similar in severity to investigator-reported CTRs.

Only four adverse events (2.9% of 136 subjects) were reported, of which two were considered related to the device. Only one subject experienced a device-related adverse event after periorbital injection (i.e., skin edema after brow injection) which was rated moderate and was resolved without sequelae after 28 days.

## Discussion

R2 has been used for over a decade, initially gaining approval for treating the under-eye area including the tear trough, where it has consistently demonstrated favorable efficacy and safety outcomes [12, 13]. Although the main indication of R2 is the tear trough, the intended use of a product can change following several years on the market [15]. Physician feedback and product usage monitoring have shown that R2 application extends beyond the tear trough to the entire periorbital region, namely the palpebromalar groove, crow’s feet, outer canthus, and brow. This prospective observational study was designed to substantiate these findings and collect robust, real-world clinical data on the extended use of R2.

EYELIGHT showed that over 70% of subjects experienced improvement in GAIS at 3 months post-injection, confirming that the primary effectiveness endpoint was met. Sensitivity analysis using an extended time window for GAIS score collection confirmed aesthetic improvement after R2 for all five periorbital indications. Subject self-assessment of GAIS scores was lower compared to the investigator, who rated over 90% of the subjects as ‘improved’ at month 3. This discrepancy could possibly arise from a difference in expectations between investigators and subjects. Subjects may hold higher expectations for the treatment than investigators [16], who possess an understanding of the realistic outcome for periorbital fillers. Additionally, subjects see the outcome every day and are likely to forget how the region looked prior to dermal filler injection and may let this influence their score, while investigators usually measure against standardized before and after pictures [17].

A high level of subject satisfaction was observed, particularly for the tear trough, palpebromalar groove, and outer canthus. For these three indications, over 80% of subjects were ‘somewhat likely’ or ‘likely’ to recommend R2. While satisfaction was lower for the brow, the majority of subjects were still satisfied with R2 and would consider recommending the product. Additionally, for all periorbital indications, most subjects required just one treatment with R2 per indication.

The study reported only one device-related AE, specifically a case of edema, which was not considered serious. These findings are similar to those of previous studies that report a low incidence of adverse events following R2 injection [12, 13]. While many AEs, such as edema, typically resolve shortly after treatment, some patients may experience prolonged or delayed reactions that can significantly impact their aesthetic outcomes, overall satisfaction, and general well-being [18]. For instance, studies have shown that complications like edema can persist even years after HA filler injection, although they are generally considered rare [19, 20]. By selecting longer follow-up periods during clinical trials, practitioners can gather pertinent data on this rare but significant complication, ultimately enhancing patient safety and informing best practices in aesthetic treatments.

CTRs occurrence was consistent with what is expected following HA filler injection [21, 22]. All CTRs were considered mild or moderate and resolved within one month of treatment. Pain during injection was common but considered mild in most cases. Even in subjects that reported severe pain, it was short-lived and had improved or resolved by the end of the visit. Real-world evidence, thus, confirmed the good safety profile of R2 in the whole periorbital region.

The EYELIGHT study allowed subjects to undergo treatment for new indications at any point throughout the study, so real-world data could be collected on many injections relative to the population size. The observational nature of the EYELIGHT study design ensured the collection of routine evidence regarding the usage of R2 beyond its initially approved indication, providing insights into the actual utilization of the product in clinical use. Furthermore, it demonstrates that registries are powerful tools to provide valuable information on the entire treatment journey, as well as factors that affect treatment outcomes [23].

Interestingly, data from the EYELIGHT study described the treatment of several indications per subject throughout the study period. This aligns with previous data from the GRADUAL study showing that subjects tend to undergo treatments for multiple indications [14], as well as previous findings that subjects and practitioners are interested in enhancing a variety of facial features [5]. These findings demonstrate an inclination toward a comprehensive treatment strategy to enhance certain facial features, ultimately striving for the best possible outcomes.

One of the inherent limitations of observational studies is the high variability of subjects and products, which would be more controlled in a randomized clinical trial, potentially generating a lower level of comparable evidence. However, well-designed observational registries, with appropriate inclusion and exclusion criteria, provide a broad representation of the usual patient population and do not overestimate or underestimate effects [24]. Although the heterogeneity of the population may introduce numerous variables, it also mirrors standard clinical practice, where practitioners make decisions regarding treatment indications, product selection, and injection techniques based on the unique needs of each patient.

Another limitation is that, despite generating substantial amounts of data, observational studies may exhibit gaps that require an appropriate statistical analysis approach [25]. Endpoints evaluated at optional follow-up visits may be more likely to result in missing data. The latter can be addressed by using appropriate sensitivity analyses, to ensure that data are filled in where possible, as was implemented in the current study. Despite these potential limitations, observational studies can provide useful real-world evidence into the routine use, efficacy, and safety of dermal fillers and can contribute with essential data to complement findings from randomized controlled trials [26].

## Conclusion

An observational design was adopted to monitor the routine use, efficacy, and safety of R2 in clinical practice and to gather real-world evidence from 136 subjects injected in, at least, one of five periorbital indications.

The EYELIGHT study demonstrated the routine use of R2 beyond its initially licensed use in the tear trough, namely in the palpebromalar groove, crow’s feet, outer canthus and brow. It unveiled a substantial proportion of subjects reporting aesthetic improvement, both subjectively and as assessed by the investigator, at 3 months post-treatment. The observation that investigators and subjects continued to perceive the subjects as ‘improved’ after 12 months, and that the number of touch-ups and retreatments was low, demonstrates the durability of R2. A low occurrence of adverse events with no device- or procedure-related serious adverse events reported, as well as the fact that all CTR were mild to moderate and transient, confirms the good safety profile of R2 in the periorbital region for a follow-up period of up to 12 months.

Taken together, these findings confirm the decade long clinical success of R2 for tear trough treatment, while establishing the efficacy and safety of R2 in addressing a variety of periorbital concerns, including the palpebromalar groove, crow’s feet, outer canthus and brow.
